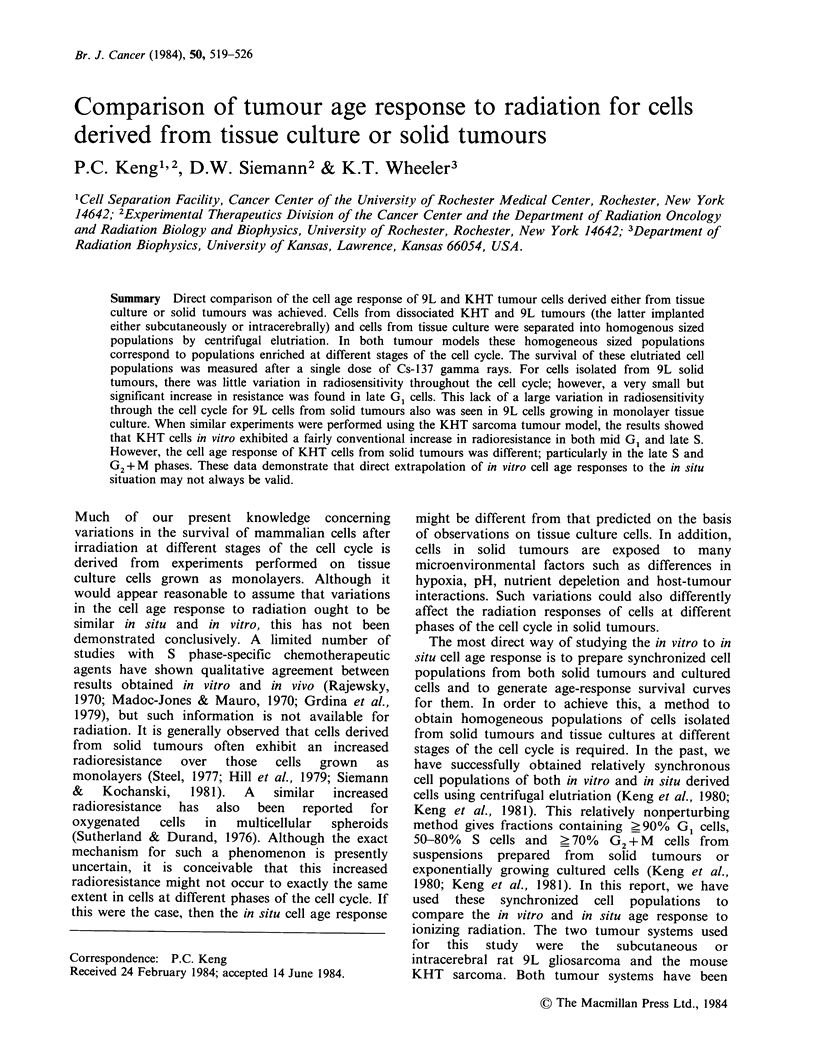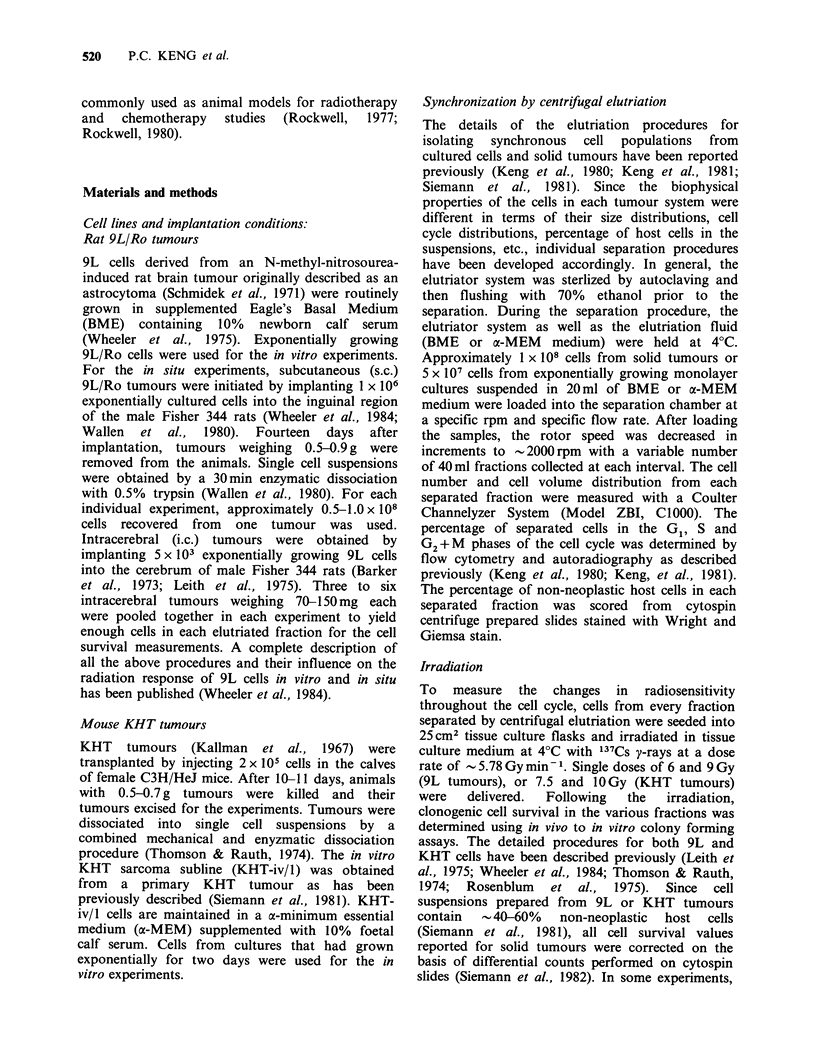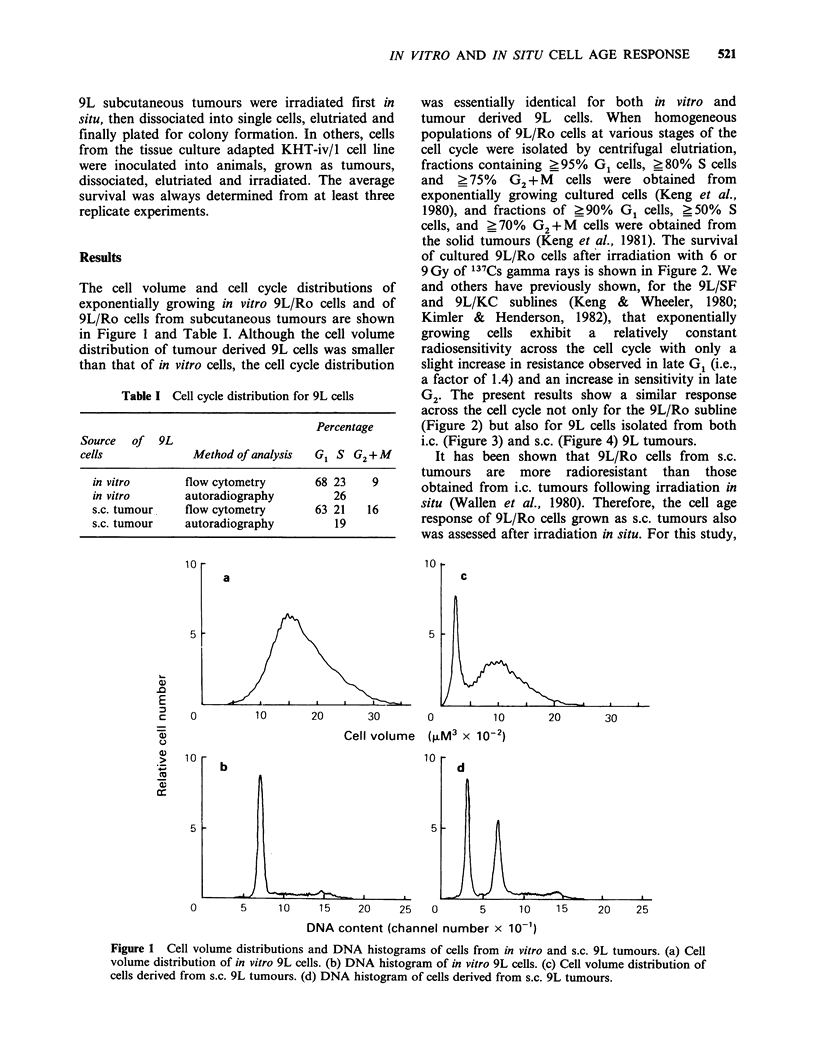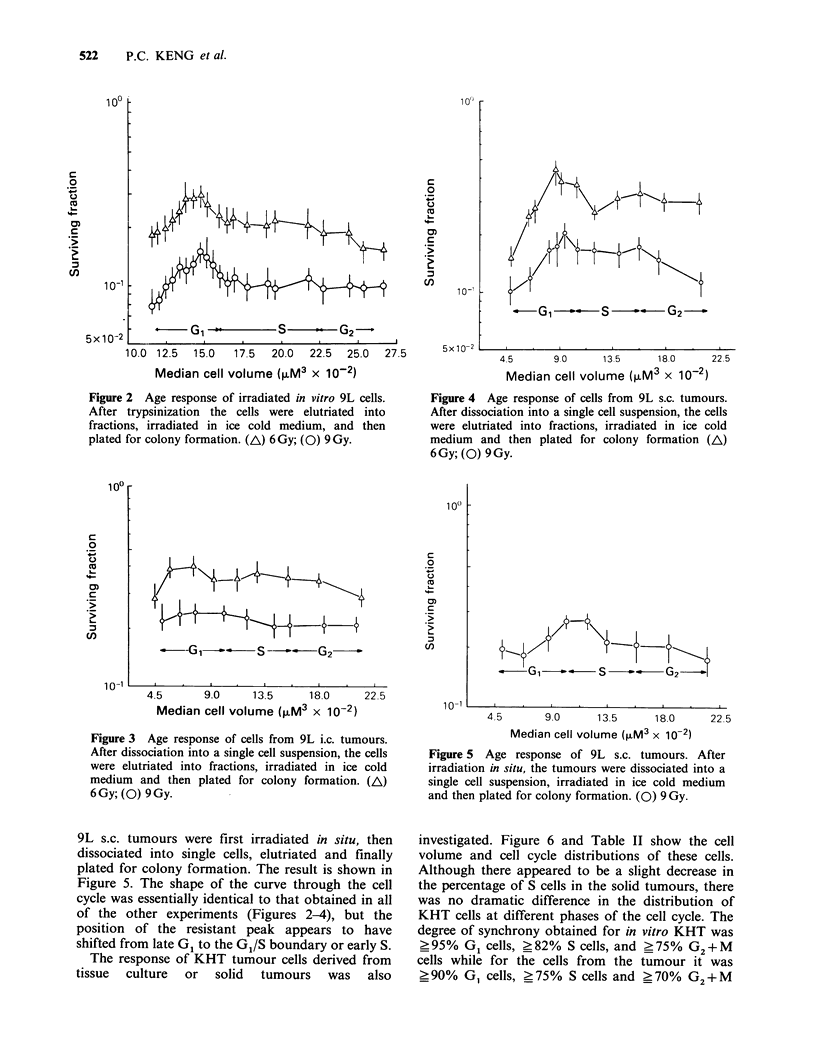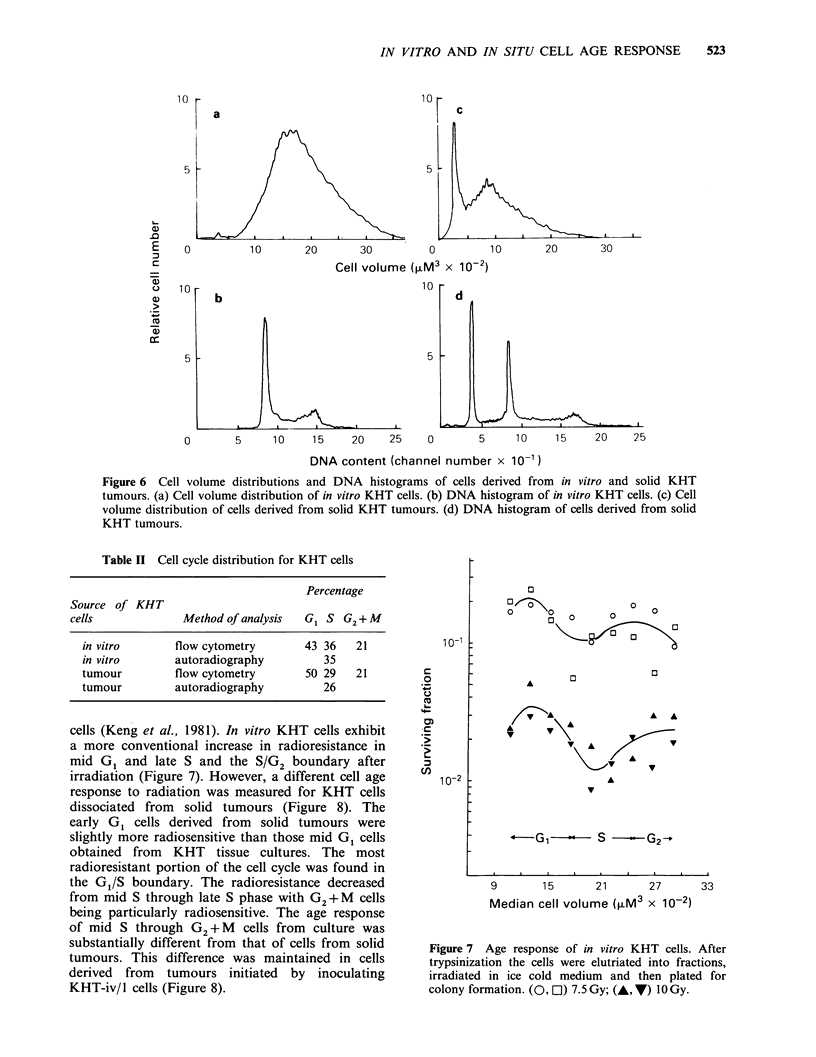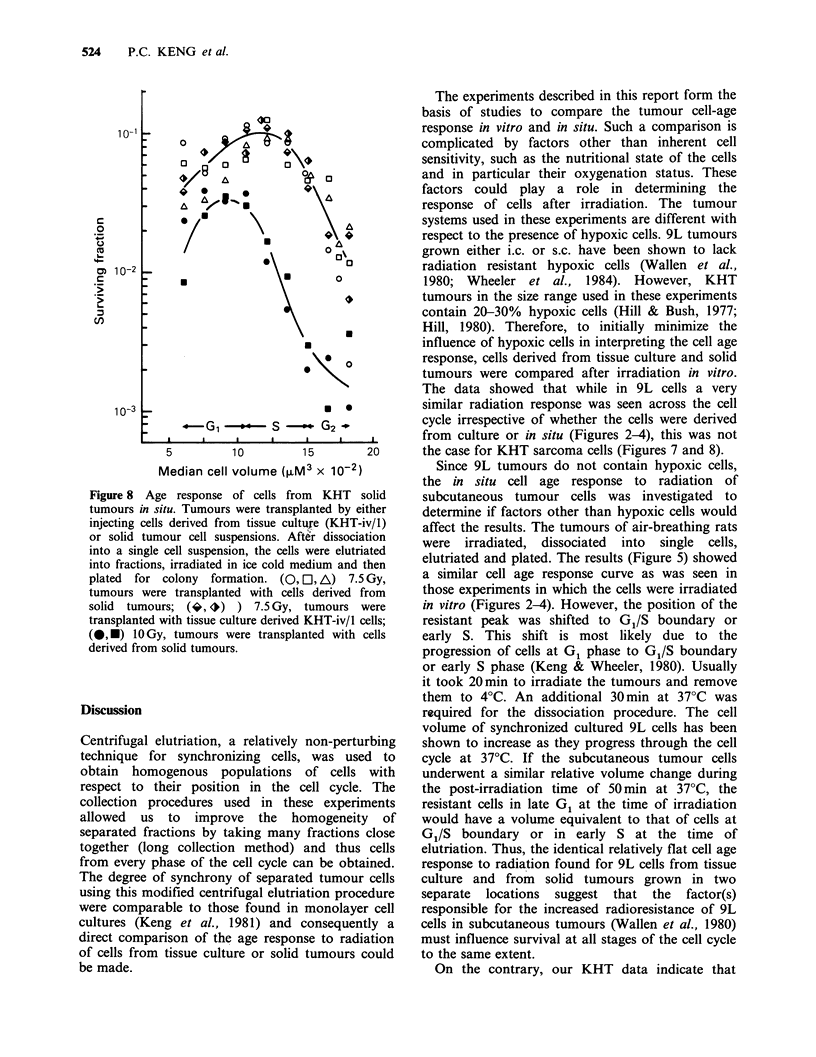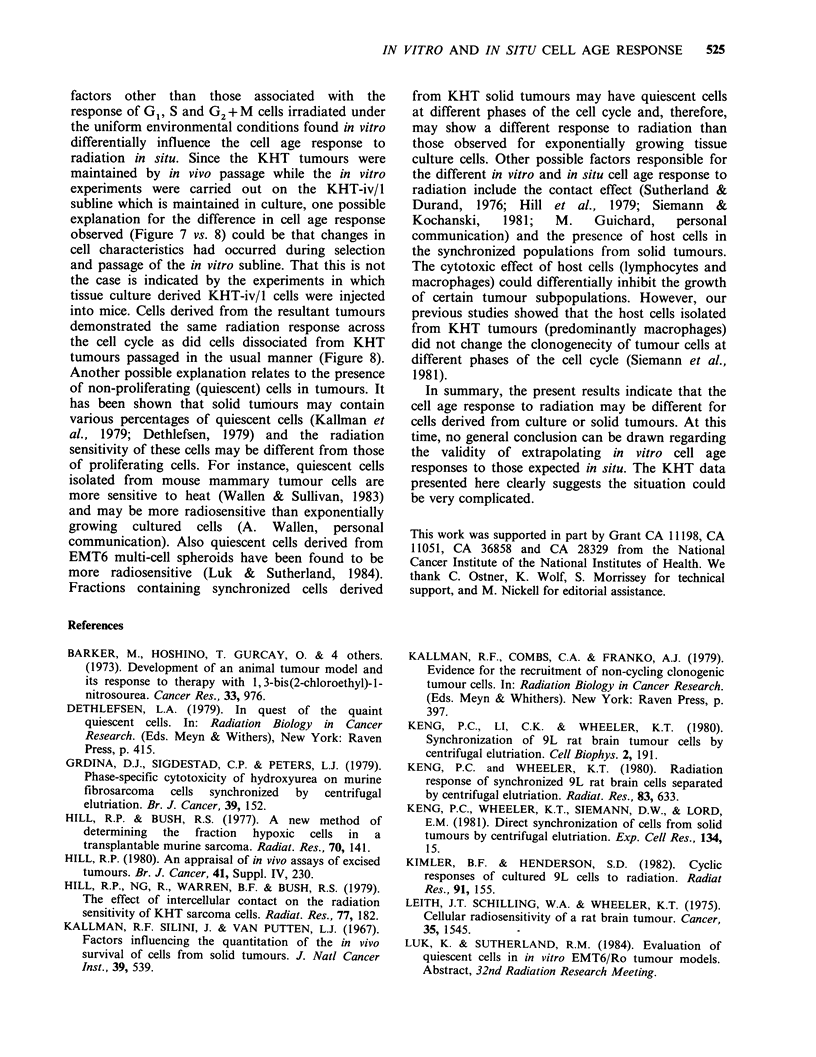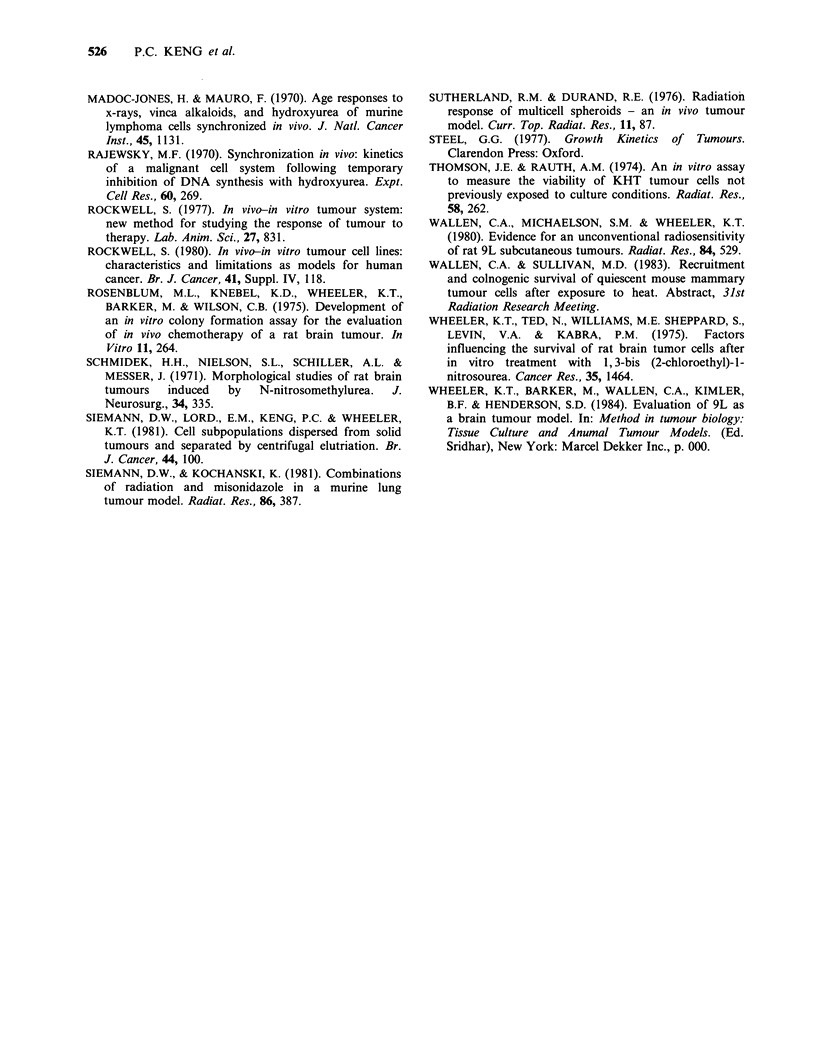# Comparison of tumour age response to radiation for cells derived from tissue culture or solid tumours.

**DOI:** 10.1038/bjc.1984.209

**Published:** 1984-10

**Authors:** P. C. Keng, D. W. Siemann, K. T. Wheeler

## Abstract

Direct comparison of the cell age response of 9L and KHT tumour cells derived either from tissue culture or solid tumours was achieved. Cells from dissociated KHT and 9L tumours (the latter implanted either subcutaneously or intracerebrally) and cells from tissue culture were separated into homogeneous sized populations by centrifugal elutriation. In both tumour models these homogeneous sized populations correspond to populations enriched at different stages of the cell cycle. The survival of these elutriated cell populations was measured after a single dose of Cs-137 gamma rays. For cells isolated from 9L solid tumours, there was little variation in radiosensitivity throughout the cell cycle; however, a very small but significant increase in resistance was found in late G1 cells. This lack of a large variation in radiosensitivity through the cell cycle for 9L cells from solid tumours also was seen in 9L cells growing in monolayer tissue culture. When similar experiments were performed using the KHT sarcoma tumour model, the results showed that KHT cells in vitro exhibited a fairly conventional increase in radioresistance in both mid G1 and late S. However, the cell age response of KHT cells from solid tumours was different; particularly in the late S and G2 + M phases. These data demonstrate that direct extrapolation of in vitro cell age responses to the in situ situation may not always be valid.


*Br. J. Cancer* (1984), **50**, 519–526

### Comparison of tumour age response to radiation for cells derived from tissue culture or solid tumours

P.C. Keng[1,2], D.W. Siemann[2] & K.T. Wheeler[3]

[1]*Cell Separation Facility, Cancer Center of the University of Rochester Medical Center, Rochester, New York 14642;* [2]*Experimental Therapeutics Division of the Cancer Center and the Department of Radiation Oncology and Radiation Biology and Biophysics, University of Rochester, Rochester, New York 14642;* [3]*Department of Radiation Biophysics, University of Kansas, Lawrence, Kansas 66054, USA.*

**Summary** Direct comparison of the cell age response of 9L and KHT tumour cells derived either from tissue culture or solid tumours was achieved. Cells from dissociated KHT and 9L tumours (the latter implanted either subcutaneously or intracerebrally) and cells from tissue culture were separated into homogenous sized populations by centrifugal elutriation. In both tumour models these homogeneous sized populations correspond to populations enriched at different stages of the cell cycle. The survival of these elutriated cell populations was measured after a single dose of Cs-137 gamma rays. For cells isolated from 9L solid tumours, there was little variation in radiosensitivity throughout the cell cycle; however, a very small but significant increase in resistance was found in late $G_1$ cells. This lack of a large variation in radiosensitivity through the cell cycle for 9L cells from solid tumours also was seen in 9L cells growing in monolayer tissue culture. When similar experiments were performed using the KHT sarcoma tumour model, the results showed that KHT cells *in vitro* exhibited a fairly conventional increase in radioresistance in both mid $G_1$ and late S. However, the cell age response of KHT cells from solid tumours was different; particularly in the late S and $G_2+M$ phases. These data demonstrate that direct extrapolation of *in vitro* cell age responses to the *in situ* situation may not always be valid.

Much of our present knowledge concerning variations in the survival of mammalian cells after irradiation at different stages of the cell cycle is derived from experiments performed on tissue culture cells grown as monolayers. Although it would appear reasonable to assume that variations in the cell age response to radiation ought to be similar *in situ* and *in vitro,* this has not been demonstrated conclusively. A limited number of studies with S phase-specific chemotherapeutic agents have shown qualitative agreement between results obtained *in vitro* and *in vivo* (Rajewsky, 1970; Madoc-Jones & Mauro, 1970; Grdina *et al.*, 1979), but such information is not available for radiation. It is generally observed that cells derived from solid tumours often exhibit an increased radioresistance over those cells grown as monolayers (Steel, 1977; Hill *et al.*, 1979; Siemann & Kochanski, 1981). A similar increased radioresistance has also been reported for oxygenated cells in multicellular spheroids (Sutherland & Durand, 1976). Although the exact mechanism for such a phenomenon is presently uncertain, it is conceivable that this increased radioresistance might not occur to exactly the same extent in cells at different phases of the cell cycle. If this were the case, then the *in situ* cell age response might be different from that predicted on the basis of observations on tissue culture cells. In addition, cells in solid tumours are exposed to many microenvironmental factors such as differences in hypoxia, pH, nutrient depeletion and host-tumour interactions. Such variations could also differently affect the radiation responses of cells at different phases of the cell cycle in solid tumours.

The most direct way of studying the *in vitro* to *in situ* cell age response is to prepare synchronized cell populations from both solid tumours and cultured cells and to generate age-response survival curves for them. In order to achieve this, a method to obtain homogeneous populations of cells isolated from solid tumours and tissue cultures at different stages of the cell cycle is required. In the past, we have successfully obtained relatively synchronous cell populations of both *in vitro* and *in situ* derived cells using centrifugal elutriation (Keng *et al.*, 1980; Keng *et al.*, 1981). This relatively nonperturbing method gives fractions containing $\geq 90\%$ $G_1$ cells, 50–80% S cells and $\geq 70\%$ $G_2+M$ cells from suspensions prepared from solid tumours or exponentially growing cultured cells (Keng *et al.*, 1980; Keng *et al.*, 1981). In this report, we have used these synchronized cell populations to compare the *in vitro* and *in situ* age response to ionizing radiation. The two tumour systems used for this study were the subcutaneous or intracerebral rat 9L gliosarcoma and the mouse KHT sarcoma. Both tumour systems have been

Correspondence: P.C. Keng
Received 24 February 1984; accepted 14 June 1984.

© The Macmillan Press Ltd., 1984

commonly used as animal models for radiotherapy and chemotherapy studies (Rockwell, 1977; Rockwell, 1980).

### Materials and methods

### *Cell lines and implantation conditions: Rat 9L/Ro tumours*

9L cells derived from an N-methyl-nitrosourea-induced rat brain tumour originally described as an astrocytoma (Schmidek *et al.*, 1971) were routinely grown in supplemented Eagle's Basal Medium (BME) containing 10% newborn calf serum (Wheeler *et al.*, 1975). Exponentially growing 9L/Ro cells were used for the *in vitro* experiments. For the *in situ* experiments, subcutaneous (s.c.) 9L/Ro tumours were initiated by implanting $1 \times 10^6$ exponentially cultured cells into the inguinal region of the male Fisher 344 rats (Wheeler *et al.*, 1984; Wallen *et al.*, 1980). Fourteen days after implantation, tumours weighing 0.5–0.9 g were removed from the animals. Single cell suspensions were obtained by a 30 min enzymatic dissociation with 0.5% trypsin (Wallen *et al.*, 1980). For each individual experiment, approximately $0.5–1.0 \times 10^8$ cells recovered from one tumour was used. Intracerebral (i.c.) tumours were obtained by implanting $5 \times 10^3$ exponentially growing 9L cells into the cerebrum of male Fisher 344 rats (Barker *et al.*, 1973; Leith *et al.*, 1975). Three to six intracerebral tumours weighing 70–150 mg each were pooled together in each experiment to yield enough cells in each elutriated fraction for the cell survival measurements. A complete description of all the above procedures and their influence on the radiation response of 9L cells *in vitro* and *in situ* has been published (Wheeler *et al.*, 1984).

### *Mouse KHT tumours*

KHT tumours (Kallman *et al.*, 1967) were transplanted by injecting $2 \times 10^5$ cells in the calves of female C3H/HeJ mice. After 10–11 days, animals with 0.5–0.7 g tumours were killed and their tumours excised for the experiments. Tumours were dissociated into single cell suspensions by a combined mechanical and enyzmatic dissociation procedure (Thomson & Rauth, 1974). The *in vitro* KHT sarcoma subline (KHT-iv/1) was obtained from a primary KHT tumour as has been previously described (Siemann *et al.*, 1981). KHT-iv/1 cells are maintained in a α-minimum essential medium (α-MEM) supplemented with 10% foetal calf serum. Cells from cultures that had grown exponentially for two days were used for the *in vitro* experiments.

### *Synchronization by centrifugal elutriation*

The details of the elutriation procedures for isolating synchronous cell populations from cultured cells and solid tumours have been reported previously (Keng *et al.*, 1980; Keng *et al.*, 1981; Siemann *et al.*, 1981). Since the biophysical properties of the cells in each tumour system were different in terms of their size distributions, cell cycle distributions, percentage of host cells in the suspensions, etc., individual separation procedures have been developed accordingly. In general, the elutriator system was sterlized by autoclaving and then flushing with 70% ethanol prior to the separation. During the separation procedure, the elutriator system as well as the elutriation fluid (BME or α-MEM medium) were held at 4°C. Approximately $1 \times 10^8$ cells from solid tumours or $5 \times 10^7$ cells from exponentially growing monolayer cultures suspended in 20 ml of BME or α-MEM medium were loaded into the separation chamber at a specific rpm and specific flow rate. After loading the samples, the rotor speed was decreased in increments to ~2000 rpm with a variable number of 40 ml fractions collected at each interval. The cell number and cell volume distribution from each separated fraction were measured with a Coulter Channelyzer System (Model ZBI, C1000). The percentage of separated cells in the $G_1$, S and $G_2+M$ phases of the cell cycle was determined by flow cytometry and autoradiography as described previously (Keng *et al.*, 1980; Keng, *et al.*, 1981). The percentage of non-neoplastic host cells in each separated fraction was scored from cytospin centrifuge prepared slides stained with Wright and Giemsa stain.

### *Irradiation*

To measure the changes in radiosensitivity throughout the cell cycle, cells from every fraction separated by centrifugal elutriation were seeded into 25 $cm^2$ tissue culture flasks and irradiated in tissue culture medium at 4°C with $^{137}Cs$ γ-rays at a dose rate of ~5.78 Gy $min^{-1}$. Single doses of 6 and 9 Gy (9L tumours), or 7.5 and 10 Gy (KHT tumours) were delivered. Following the irradiation, clonogenic cell survival in the various fractions was determined using *in vivo* to *in vitro* colony forming assays. The detailed procedures for both 9L and KHT cells have been described previously (Leith *et al.*, 1975; Wheeler *et al.*, 1984; Thomson & Rauth, 1974; Rosenblum *et al.*, 1975). Since cell suspensions prepared from 9L or KHT tumours contain ~40–60% non-neoplastic host cells (Siemann *et al.*, 1981), all cell survival values reported for solid tumours were corrected on the basis of differential counts performed on cytospin slides (Siemann *et al.*, 1982). In some experiments,

9L subcutaneous tumours were irradiated first *in situ*, then dissociated into single cells, elutriated and finally plated for colony formation. In others, cells from the tissue culture adapted KHT-iv/1 cell line were inoculated into animals, grown as tumours, dissociated, elutriated and irradiated. The average survival was always determined from at least three replicate experiments.

### Results

The cell volume and cell cycle distributions of exponentially growing *in vitro* 9L/Ro cells and of 9L/Ro cells from subcutaneous tumours are shown in Figure 1 and Table I. Although the cell volume distribution of tumour derived 9L cells was smaller than that of *in vitro* cells, the cell cycle distribution was essentially identical for both *in vitro* and tumour derived 9L cells. When homogeneous populations of 9L/Ro cells at various stages of the cell cycle were isolated by centrifugal elutriation, fractions containing $\geq$95% $G_1$ cells, $\geq$80% S cells and $\geq$75% $G_2$+M cells were obtained from exponentially growing cultured cells (Keng *et al.*, 1980), and fractions of $\geq$90% $G_1$ cells, $\geq$50% S cells, and $\geq$70% $G_2$+M cells were obtained from the solid tumours (Keng *et al.*, 1981). The survival of cultured 9L/Ro cells after irradiation with 6 or 9 Gy of $^{137}$Cs gamma rays is shown in Figure 2. We and others have previously shown, for the 9L/SF and 9L/KC sublines (Keng & Wheeler, 1980; Kimler & Henderson, 1982), that exponentially growing cells exhibit a relatively constant radiosensitivity across the cell cycle with only a slight increase in resistance observed in late $G_1$ (i.e., a factor of 1.4) and an increase in sensitivity in late $G_2$. The present results show a similar response across the cell cycle not only for the 9L/Ro subline (Figure 2) but also for 9L cells isolated from both i.c. (Figure 3) and s.c. (Figure 4) 9L tumours.

It has been shown that 9L/Ro cells from s.c. tumours are more radioresistant than those obtained from i.c. tumours following irradiation *in situ* (Wallen *et al.*, 1980). Therefore, the cell age response of 9L/Ro cells grown as s.c. tumours also was assessed after irradiation *in situ*. For this study,

**Table I** Cell cycle distribution for 9L cells

| Source of 9L cells | Method of analysis | Percentage $G_1$ | $S$ | $G_2$+$M$ |
|---|---|---|---|---|
| *in vitro* | flow cytometry | 68 | 23 | 9 |
| *in vitro* | autoradiography | | 26 | |
| s.c. tumour | flow cytometry | 63 | 21 | 16 |
| s.c. tumour | autoradiography | | 19 | |

**Figure 1** Cell volume distributions and DNA histograms of cells from *in vitro* and s.c. 9L tumours. (a) Cell volume distribution of *in vitro* 9L cells. (b) DNA histogram of *in vitro* 9L cells. (c) Cell volume distribution of cells derived from s.c. 9L tumours. (d) DNA histogram of cells derived from s.c. 9L tumours.

**Figure 2** Age response of irradiated *in vitro* 9L cells. After trypsinization the cells were elutriated into fractions, irradiated in ice cold medium, and then plated for colony formation. (△) 6 Gy; (○) 9 Gy.

**Figure 4** Age response of cells from 9L s.c. tumours. After dissociation into a single cell suspension, the cells were elutriated into fractions, irradiated in ice cold medium and then plated for colony formation (△) 6 Gy; (○) 9 Gy.

**Figure 3** Age response of cells from 9L i.c. tumours. After dissociation into a single cell suspension, the cells were elutriated into fractions, irradiated in ice cold medium and then plated for colony formation. (△) 6 Gy; (○) 9 Gy.

**Figure 5** Age response of 9L s.c. tumours. After irradiation *in situ*, the tumours were dissociated into a single cell suspension, irradiated in ice cold medium and then plated for colony formation. (○) 9 Gy.

9L s.c. tumours were first irradiated *in situ*, then dissociated into single cells, elutriated and finally plated for colony formation. The result is shown in Figure 5. The shape of the curve through the cell cycle was essentially identical to that obtained in all of the other experiments (Figures 2–4), but the position of the resistant peak appears to have shifted from late $G_1$ to the $G_1$/S boundary or early S.

The response of KHT tumour cells derived from tissue culture or solid tumours was also investigated. Figure 6 and Table II show the cell volume and cell cycle distributions of these cells. Although there appeared to be a slight decrease in the percentage of S cells in the solid tumours, there was no dramatic difference in the distribution of KHT cells at different phases of the cell cycle. The degree of synchrony obtained for *in vitro* KHT was $\geq$95% $G_1$ cells, $\geq$82% S cells, and $\geq$75% $G_2$+M cells while for the cells from the tumour it was $\geq$90% $G_1$ cells, $\geq$75% S cells and $\geq$70% $G_2$+M

**Figure 6** Cell volume distributions and DNA histograms of cells derived from *in vitro* and solid KHT tumours. (a) Cell volume distribution of *in vitro* KHT cells. (b) DNA histogram of *in vitro* KHT cells. (c) Cell volume distribution of cells derived from solid KHT tumours. (d) DNA histogram of cells derived from solid KHT tumours.

**Table II** Cell cycle distribution for KHT cells

| *Source of KHT cells* | *Method of analysis* | *Percentage* $G_1$ | $S$ | $G_2+M$ |
|---|---|---|---|---|
| *in vitro* | flow cytometry | 43 | 36 | 21 |
| *in vitro* | autoradiography | | 35 | |
| tumour | flow cytometry | 50 | 29 | 21 |
| tumour | autoradiography | | 26 | |

cells (Keng *et al.*, 1981). *In vitro* KHT cells exhibit a more conventional increase in radioresistance in mid $G_1$ and late S and the $S/G_2$ boundary after irradiation (Figure 7). However, a different cell age response to radiation was measured for KHT cells dissociated from solid tumours (Figure 8). The early $G_1$ cells derived from solid tumours were slightly more radiosensitive than those mid $G_1$ cells obtained from KHT tissue cultures. The most radioresistant portion of the cell cycle was found in the $G_1/S$ boundary. The radioresistance decreased from mid S through late S phase with $G_2+M$ cells being particularly radiosensitive. The age response of mid S through $G_2+M$ cells from culture was substantially different from that of cells from solid tumours. This difference was maintained in cells derived from tumours initiated by inoculating KHT-iv/1 cells (Figure 8).

**Figure 7** Age response of *in vitro* KHT cells. After trypsinization the cells were elutriated into fractions, irradiated in ice cold medium and then plated for colony formation. (○, □) 7.5 Gy; (▲, ▼) 10 Gy.

**Figure 8** Age response of cells from KHT solid tumours *in situ*. Tumours were transplanted by either injecting cells derived from tissue culture (KHT-iv/1) or solid tumour cell suspensions. After dissociation into a single cell suspension, the cells were elutriated into fractions, irradiated in ice cold medium and then plated for colony formation. (○, □, △) 7.5 Gy, tumours were transplanted with cells derived from solid tumours; (◆, ◆) ) 7.5 Gy, tumours were transplanted with tissue culture derived KHT-iv/1 cells; (●, ■) 10 Gy, tumours were transplanted with cells derived from solid tumours.

### Discussion

Centrifugal elutriation, a relatively non-perturbing technique for synchronizing cells, was used to obtain homogenous populations of cells with respect to their position in the cell cycle. The collection procedures used in these experiments allowed us to improve the homogeneity of separated fractions by taking many fractions close together (long collection method) and thus cells from every phase of the cell cycle can be obtained. The degree of synchrony of separated tumour cells using this modified centrifugal elutriation procedure were comparable to those found in monolayer cell cultures (Keng *et al.*, 1981) and consequently a direct comparison of the age response to radiation of cells from tissue culture or solid tumours could be made.

The experiments described in this report form the basis of studies to compare the tumour cell-age response *in vitro* and *in situ*. Such a comparison is complicated by factors other than inherent cell sensitivity, such as the nutritional state of the cells and in particular their oxygenation status. These factors could play a role in determining the response of cells after irradiation. The tumour systems used in these experiments are different with respect to the presence of hypoxic cells. 9L tumours grown either i.c. or s.c. have been shown to lack radiation resistant hypoxic cells (Wallen *et al.*, 1980; Wheeler *et al.*, 1984). However, KHT tumours in the size range used in these experiments contain 20–30% hypoxic cells (Hill & Bush, 1977; Hill, 1980). Therefore, to initially minimize the influence of hypoxic cells in interpreting the cell age response, cells derived from tissue culture and solid tumours were compared after irradiation *in vitro*. The data showed that while in 9L cells a very similar radiation response was seen across the cell cycle irrespective of whether the cells were derived from culture or *in situ* (Figures 2–4), this was not the case for KHT sarcoma cells (Figures 7 and 8).

Since 9L tumours do not contain hypoxic cells, the *in situ* cell age response to radiation of subcutaneous tumour cells was investigated to determine if factors other than hypoxic cells would affect the results. The tumours of air-breathing rats were irradiated, dissociated into single cells, elutriated and plated. The results (Figure 5) showed a similar cell age response curve as was seen in those experiments in which the cells were irradiated *in vitro* (Figures 2–4). However, the position of the resistant peak was shifted to $G_1$/S boundary or early S. This shift is most likely due to the progression of cells at $G_1$ phase to $G_1$/S boundary or early S phase (Keng & Wheeler, 1980). Usually it took 20 min to irradiate the tumours and remove them to 4°C. An additional 30 min at 37°C was required for the dissociation procedure. The cell volume of synchronized cultured 9L cells has been shown to increase as they progress through the cell cycle at 37°C. If the subcutaneous tumour cells underwent a similar relative volume change during the post-irradiation time of 50 min at 37°C, the resistant cells in late $G_1$ at the time of irradiation would have a volume equivalent to that of cells at $G_1$/S boundary or in early S at the time of elutriation. Thus, the identical relatively flat cell age response to radiation found for 9L cells from tissue culture and from solid tumours grown in two separate locations suggest that the factor(s) responsible for the increased radioresistance of 9L cells in subcutaneous tumours (Wallen *et al.*, 1980) must influence survival at all stages of the cell cycle to the same extent.

On the contrary, our KHT data indicate that factors other than those associated with the response of $G_1$, S and $G_2+M$ cells irradiated under the uniform environmental conditions found *in vitro* differentially influence the cell age response to radiation *in situ*. Since the KHT tumours were maintained by *in vivo* passage while the *in vitro* experiments were carried out on the KHT-iv/1 subline which is maintained in culture, one possible explanation for the difference in cell age response observed (Figure 7 *vs.* 8) could be that changes in cell characteristics had occurred during selection and passage of the *in vitro* subline. That this is not the case is indicated by the experiments in which tissue culture derived KHT-iv/1 cells were injected into mice. Cells derived from the resultant tumours demonstrated the same radiation response across the cell cycle as did cells dissociated from KHT tumours passaged in the usual manner (Figure 8). Another possible explanation relates to the presence of non-proliferating (quiescent) cells in tumours. It has been shown that solid tumours may contain various percentages of quiescent cells (Kallman *et al.*, 1979; Dethlefsen, 1979) and the radiation sensitivity of these cells may be different from those of proliferating cells. For instance, quiescent cells isolated from mouse mammary tumour cells are more sensitive to heat (Wallen & Sullivan, 1983) and may be more radiosensitive than exponentially growing cultured cells (A. Wallen, personal communication). Also quiescent cells derived from EMT6 multi-cell spheroids have been found to be more radiosensitive (Luk & Sutherland, 1984). Fractions containing synchronized cells derived from KHT solid tumours may have quiescent cells at different phases of the cell cycle and, therefore, may show a different response to radiation than those observed for exponentially growing tissue culture cells. Other possible factors responsible for the different *in vitro* and *in situ* cell age response to radiation include the contact effect (Sutherland & Durand, 1976; Hill *et al.*, 1979; Siemann & Kochanski, 1981; M. Guichard, personal communication) and the presence of host cells in the synchronized populations from solid tumours. The cytotoxic effect of host cells (lymphocytes and macrophages) could differentially inhibit the growth of certain tumour subpopulations. However, our previous studies showed that the host cells isolated from KHT tumours (predominantly macrophages) did not change the clonogenecity of tumour cells at different phases of the cell cycle (Siemann *et al.*, 1981).

In summary, the present results indicate that the cell age response to radiation may be different for cells derived from culture or solid tumours. At this time, no general conclusion can be drawn regarding the validity of extrapolating *in vitro* cell age responses to those expected *in situ*. The KHT data presented here clearly suggests the situation could be very complicated.

This work was supported in part by Grant CA 11198, CA 11051, CA 36858 and CA 28329 from the National Cancer Institute of the National Institutes of Health. We thank C. Ostner, K. Wolf, S. Morrissey for technical support, and M. Nickell for editorial assistance.

### References

BARKER, M., HOSHINO, T. GURCAY, O. & 4 others. (1973). Development of an animal tumour model and its response to therapy with 1,3-bis(2-chloroethyl)-1-nitrosourea. *Cancer Res.*, **33**, 976.

DETHLEFSEN, L.A. (1979). In quest of the quaint quiescent cells. In: *Radiation Biology in Cancer Research*. (Eds. Meyn & Withers), New York: Raven Press, p. 415.

GRDINA, D.J., SIGDESTAD, C.P. & PETERS, L.J. (1979). Phase-specific cytotoxicity of hydroxyurea on murine fibrosarcoma cells synchronized by centrifugal elutriation. *Br. J. Cancer*, **39**, 152.

HILL, R.P. & BUSH, R.S. (1977). A new method of determining the fraction hypoxic cells in a transplantable murine sarcoma. *Radiat. Res.*, **70**, 141.

HILL, R.P. (1980). An appraisal of *in vivo* assays of excised tumours. *Br. J. Cancer*, **41**, Suppl. IV, 230.

HILL, R.P., NG, R., WARREN, B.F. & BUSH, R.S. (1979). The effect of intercellular contact on the radiation sensitivity of KHT sarcoma cells. *Radiat. Res.*, **77**, 182.

KALLMAN, R.F. SILINI, J. & VAN PUTTEN, L.J. (1967). Factors influencing the quantitation of the *in vivo* survival of cells from solid tumours. *J. Natl Cancer Inst.*, **39**, 539.

KALLMAN, R.F., COMBS, C.A. & FRANKO, A.J. (1979). Evidence for the recruitment of non-cycling clonogenic tumour cells. In: *Radiation Biology in Cancer Research*. (Eds. Meyn & Whithers). New York: Raven Press, p. 397.

KENG, P.C., LI, C.K. & WHEELER, K.T. (1980). Synchronization of 9L rat brain tumour cells by centrifugal elutriation. *Cell Biophys.* **2**, 191.

KENG, P.C. and WHEELER, K.T. (1980). Radiation response of synchronized 9L rat brain cells separated by centrifugal elutriation. *Radiat. Res.*, **83**, 633.

KENG, P.C., WHEELER, K.T., SIEMANN, D.W., & LORD, E.M. (1981). Direct synchronization of cells from solid tumours by centrifugal elutriation. *Exp. Cell Res.*, **134**, 15.

KIMLER, B.F. & HENDERSON, S.D. (1982). Cyclic responses of cultured 9L cells to radiation. *Radiat Res.*, **91**, 155.

LEITH, J.T. SCHILLING, W.A. & WHEELER, K.T. (1975). Cellular radiosensitivity of a rat brain tumour. *Cancer*, **35**, 1545.

LUK, K. & SUTHERLAND, R.M. (1984). Evaluation of quiescent cells in *in vitro* EMT6/Ro tumour models. Abstract, *32nd Radiation Research Meeting*.

MADOC-JONES, H. & MAURO, F. (1970). Age responses to x-rays, vinca alkaloids, and hydroxyurea of murine lymphoma cells synchronized *in vivo*. *J. Natl. Cancer Inst.*, **45**, 1131.

RAJEWSKY, M.F. (1970). Synchronization *in vivo*: kinetics of a malignant cell system following temporary inhibition of DNA synthesis with hydroxyurea. *Expt. Cell Res.*, **60**, 269.

ROCKWELL, S. (1977). *In vivo–in vitro* tumour system: new method for studying the response of tumour to therapy. *Lab. Anim. Sci.*, **27**, 831.

ROCKWELL, S. (1980). *In vivo–in vitro* tumour cell lines: characteristics and limitations as models for human cancer. *Br. J. Cancer*, **41**, Suppl. IV, 118.

ROSENBLUM, M.L., KNEBEL, K.D., WHEELER, K.T., BARKER, M. & WILSON, C.B. (1975). Development of an *in vitro* colony formation assay for the evaluation of *in vivo* chemotherapy of a rat brain tumour. *In Vitro* **11**, 264.

SCHMIDEK, H.H., NIELSON, S.L., SCHILLER, A.L. & MESSER, J. (1971). Morphological studies of rat brain tumours induced by N-nitrosomethylurea. *J.* Neurosurg., **34**, 335.

SIEMANN, D.W., LORD., E.M., KENG, P.C. & WHEELER, K.T. (1981). Cell subpopulations dispersed from solid tumours and separated by centrifugal elutriation. *Br. J. Cancer*, **44**, 100.

SIEMANN, D.W., & KOCHANSKI, K. (1981). Combinations of radiation and misonidazole in a murine lung tumour model. *Radiat. Res.*, **86**, 387.

SUTHERLAND, R.M. & DURAND, R.E. (1976). Radiation response of multicell spheroids – an *in vivo* tumour model. *Curr. Top. Radiat. Res.*, **11**, 87.

STEEL, G.G. (1977). *Growth Kinetics of Tumours*. Clarendon Press: Oxford.

THOMSON, J.E. & RAUTH, A.M. (1974). An *in vitro* assay to measure the viability of KHT tumour cells not previously exposed to culture conditions. *Radiat. Res.*, **58**, 262.

WALLEN, C.A., MICHAELSON, S.M. & WHEELER, K.T. (1980). Evidence for an unconventional radiosensitivity of rat 9L subcutaneous tumours. *Radiat. Res.*, **84**, 529.

WALLEN, C.A. & SULLIVAN, M.D. (1983). Recruitment and colnogenic survival of quiescent mouse mammary tumour cells after exposure to heat. Abstract, *31st Radiation Research Meeting*.

WHEELER, K.T., TED, N., WILLIAMS, M.E. SHEPPARD, S., LEVIN, V.A. & KABRA, P.M. (1975). Factors influencing the survival of rat brain tumor cells after in vitro treatment with 1,3-bis (2-chloroethyl)-1-nitrosourea. *Cancer Res.*, **35**, 1464.

WHEELER, K.T., BARKER, M., WALLEN, C.A., KIMLER, B.F. & HENDERSON, S.D. (1984). Evaluation of 9L as a brain tumour model. In: *Method in tumour biology: Tissue Culture and Anumal Tumour Models*. (Ed. Sridhar), New York: Marcel Dekker Inc., p. 000.